# Temporal Regulation of Early-Stage Cytokine Expression in Diabetic Wound Healing Under Negative Pressure Wound Therapy

**DOI:** 10.3390/ijms26104634

**Published:** 2025-05-13

**Authors:** Hua-Sheng Chiu, Ting-Shuo Huang, Chien-Tzung Chen, Xin-Yu Lin, Po-Cheng Liao, Cai-Cin Liou, Chih-Chin Hsu, Sonal Somvanshi, Pavel Sumazin, Pang-Hung Hsu, Chi-Chin Sun, Yu-Chiau Shyu

**Affiliations:** 1 Department of Pediatrics, Texas Children’s Hospital Cancer Center, Baylor College of Medicine, Houston, TX 77030, USA; hua-sheng.chiu@bcm.edu (H.-S.C.); sonal.somvanshi@bcm.edu (S.S.);; 2 Department of General Surgery, Jen Ai Hospital, Taichung 400, Taiwan; huangts1234@gmail.com; 3 School of Traditional Chinese Medicine, College of Medicine, Chang Gung University, Taoyuan 333, Taiwan; 4 Department of Plastic and Reconstructive Surgery, Chang Gung Memorial Hospital, Taoyuan 333, Taiwan; ctchenap@cgmh.org.tw; 5 Craniofacial Research Center, Chang Gung University, Taoyuan 333, Taiwan; 6 Community Medicine Research Center, Chang Gung Memorial Hospital, Keelung Branch, Keelung 204, Taiwan; love20070810@gmail.com (X.-Y.L.); henryshome@gmail.com (P.-C.L.); nice302a@gmail.com (C.-C.L.); 7 School of Medicine, College of Medicine, Chang Gung University, Taoyuan 333, Taiwan; steele0618@gmail.com (C.-C.H.); chichinsun@gmail.com (C.-C.S.); 8 Department of Physical Medicine and Rehabilitation, Chang Gung Memorial Hospital, Keelung Branch, Keelung 204, Taiwan; 9 Department of Bioscience and Biotechnology, National Taiwan Ocean University, Keelung 202, Taiwan; phsu@ntou.edu.tw; 10 Department of Ophthalmology, Chang Gung Memorial Hospital, Keelung Branch, Keelung 204, Taiwan; 11 Department of Nursing, Chang Gung University of Science and Technology, Taoyuan 333, Taiwan

**Keywords:** negative pressure wound therapy (NPWT), diabetic (DB), wound healing, cytokine profiling

## Abstract

Negative pressure wound therapy (NPWT) is widely recognized for its efficacy in treating diabetic wounds, but the mechanisms involved in the wound healing process remain unclear. By examining changes in blood cytokine levels as molecular signaling precursors, we aim to provide a comprehensive cytokine profile to support adjunctive therapy research and clinical applications. A diabetic mouse wound model was established to compare cytokine profiles between NPWT-treated and standard dressing groups, identifying key signaling candidates that may facilitate wound healing. By integrating normal mouse data with large-scale cytokine analysis, we developed a time-stratified NPWT approach to track acute-phase cytokine fluctuations in diabetic conditions. NPWT did not significantly enhance coagulation-related cytokine expression but effectively reduced inflammation, albeit with a delayed regulatory effect compared to wild-type mice. A one-sided binomial test revealed that NPWT advanced the cytokine expression peak from 16 to 2 h, partially restoring the early healing pattern seen in normal mice and suggesting its potential role in modulating early-stage wound repair. These findings provide novel insights into early cytokine regulation during wound healing and highlight the potential of NPWT to inform therapeutic strategies. This refined monitoring approach may contribute to improved clinical decision-making and support enhanced wound management in diabetic patients.

## 1. Introduction

The skin, as the largest organ of the human body, plays a critical role in protecting against environmental hazards. In addition to its role as a physical and immunological barrier, the skin is involved in thermoregulation, sensory perception, storage, synthesis, and absorption [1]. It consists of three layers: the epidermis, dermis, and hypodermis, with the dermis containing collagen, elastic fibers, and various appendages [1]. Upon injury, the body initiates the production of collagen and elastic fibers to repair the wound. However, extensive injuries or those occurring in the context of chronic diseases can result in chronic wounds, infections, or even life-threatening complications [2]. Wound healing is an intricate process that relies on the coordinated action of multiple signaling pathways and molecular mechanisms. While most superficial wounds heal without complications, conditions such as diabetes can impair this process, increasing the burden on patients and healthcare systems.

Negative Pressure Wound Therapy (NPWT) has emerged as an effective approach to accelerate the healing of difficult-to-heal wounds. It reduces infection risk, minimizes dressing changes, and lowers overall wound care costs, underscoring its growing significance in clinical practice. NPWT is applicable to a variety of wounds, including surgical incisions, large skin defects, acute burn injuries, pressure ulcers, and diabetic foot ulcers (DFUs). Since 2000, numerous studies have reported the effectiveness of NPWT in enhancing wound healing, demonstrating faster healing times in patients with foot amputations and other extensive wounds [3,4]. Systematic reviews and meta-analyses confirm that NPWT significantly improves healing rates (OR = 3.60) and shortens granulation tissue formation time, reinforcing its clinical value [5]. Diabetic (DB) patients often face impaired wound healing and an increased risk of postoperative infections, particularly those with diabetes-related peripheral arterial disease undergoing amputation [6,7]. Furthermore, diabetic wounds are characterized by dysregulated cytokine expression and a prolonged inflammatory phase, contributing to delayed healing and increased susceptibility to infections [8]. Wound healing is a complex biological process involving an intricate network of cells, cytokines, and the extracellular matrix and comprises stages such as hemostasis, inflammation, proliferation, and remodeling [9].

Despite extensive research aimed at understanding the molecular mechanisms of wound healing, many studies tend to focus on isolated pathways, often lacking an integrated view of the dynamic regulation of cytokine expression, particularly under hyperglycemic conditions. In diabetic wounds, delayed production of chemokines and cytokines can impair immune cell activation, prolong the inflammatory phase, and hinder progression to later stages of healing [10,11,12]. Although NPWT has demonstrated efficacy in reducing inflammation and accelerating wound closure, a comprehensive understanding of the cytokine response to NPWT, especially in hyperglycemic conditions, remains elusive [13,14]. The persistence of regulatory mechanisms involved in wound healing under hyperglycemic conditions, alongside inflammatory responses and cytokine variations, is still not fully clarified. Prior research indicates that delayed chemokine and cytokine production in diabetic wounds leads to slower monocyte infiltration and macrophage activation [10]. This lag can impair phagocytosis, resulting in the accumulation of wound debris, apoptotic cells, and neutrophils [11]. Such debris contributes to a prolonged inflammatory phase, during which activated neutrophils release proteases that nonspecifically degrade the wound microenvironment [12]. Furthermore, diabetic wounds often exhibit inflammatory mediators such as IL-1β (secreted by M1 macrophages), nitric oxide, and inducible nitric oxide synthase (iNOS), all of which inhibit wound healing [13,14]. While NPWT has been shown to mitigate inflammation and expedite healing in hyperglycemic conditions [15,16,17], the comprehensive mechanisms by which NPWT induces chemokine and cytokine responses remain unresolved.

## 2. Results

### 2.1. Comparison of the Effects of NPWT Versus AP on 110 Cytokines Across the Four Main Stages of Wound Healing

**Figure 1:**
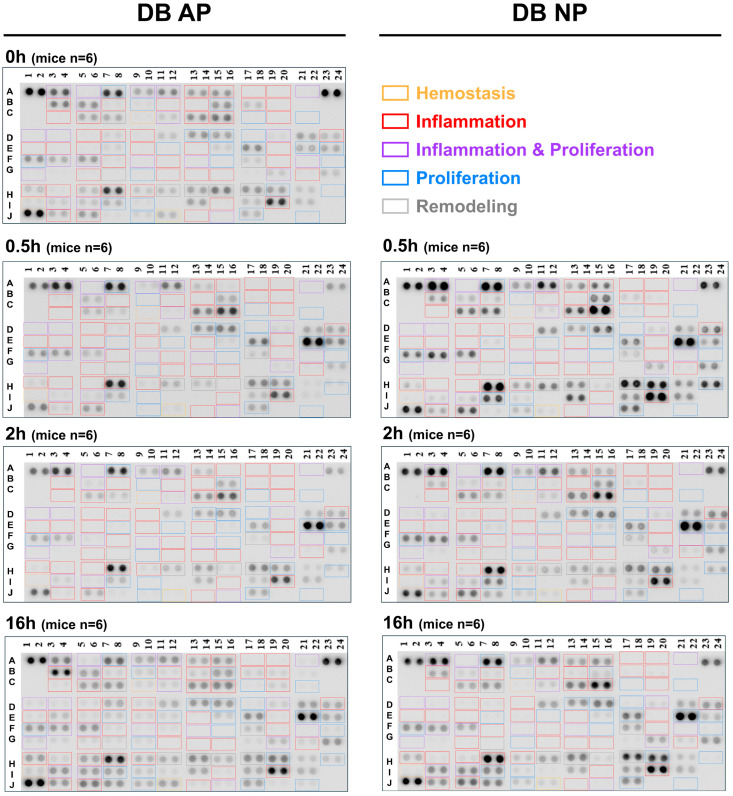
Cytokine expression in diabetic wound healing. Cytokine expression profiles in type 2 diabetic mouse model at four-time points: 0, 0.5, 2, and 16 h after wounding under two pressure conditions: AP and NP. Color frames indicate wound healing stages (early to late): hemostasis (orange), inflammation (red), inflammation and proliferation (purple), proliferation (blue), and remodeling (grey). Rows A through J represent cytokine target spots based on the predefined array layout of the Mouse XL Cytokine Array (R&D Systems). For details on spot assignments and cytokine identities, please refer to the product documentation provided by the manufacturer.

The cytokine array analysis of serum samples from diabetic (BKS.Cg-Dock7^m^+/+ Lepr^db^/JNarl) mice, treated with either AP or NP wound therapy at early time points (0, 0.5, 2, and 16 h), was conducted using the Mouse XL Cytokine Array Kit (R&D). Color-coded frames represent the stages of wound healing, progressing from early to late: orange for hemostasis, red for inflammation, purple for inflammation and proliferation, blue for proliferation, and grey for remodeling. To facilitate subsequent analysis, the four main stages of wound healing were distinguished by different colors. The inflammation stage was further subdivided into early and late phases, with additional granularity provided for the proliferation stage (Figure 1).

### 2.2. Cytokine Modulation Delayed in Diabetic Mice Under Negative Pressure Wound Therapy

In our cytokine array analysis, we examined serum samples from diabetic mice treated with AP or NP wound therapy at early time points (0, 0.5, 2, and 16 h). To ensure accuracy, we employed a well-established array normalization method that accounted for biological and technical variability. Each array included two replicates, with three reference spots (positive controls) per replicate for normalization. The total intensity of these positive controls served as the baseline for normalizing the intensities of other cytokine probes on the array. For detailed analysis, we categorized the four main stages of wound healing, with additional subdivisions for early and late inflammation, as well as for proliferation. The raw data were presented in the Supplementary Information section to allow other investigators to conduct further research.

Cytokine fold changes were determined by comparing the normalized intensities under different experimental conditions (AP or NP at 0.5, 2, or 16 h) against the control group (AP at 0 h). The probes were further grouped into five functional categories corresponding to different wound healing stages: hemostasis (3 genes), inflammation (43 genes), inflammation and proliferation (20 genes), proliferation (26 genes), and remodeling (7 genes). The total fold change for each category was calculated by summing the fold changes of all cytokines within that group.

**Figure 2:**
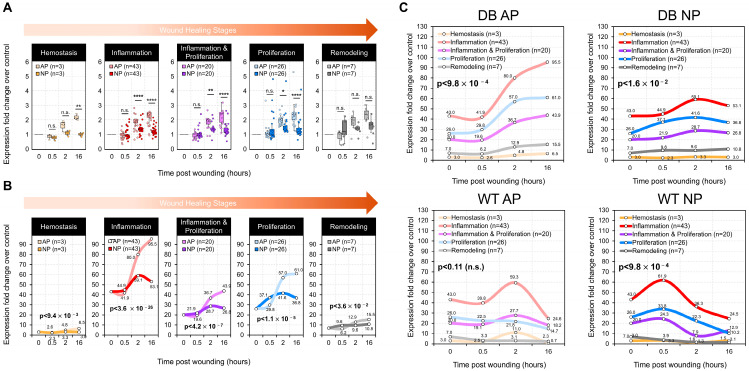
Negative pressure promotes early wound healing by downregulating cytokines. (**A**) Box plots depict fold changes in individual cytokine gene expression relative to control (AP at 0 h) across time points or wound healing stages for AP and NP groups. (**B**) Line charts show the total fold change in gene expression (summed across all genes) for each stage, comparing AP and NP groups over time. *p*-values for statistical significance of AP vs. NP expression fold change differences were calculated by Student’s *t*-tests (**A**), then combined across time points using Fisher’s method (**B**); * *p* < 0.05, ** *p* < 0.01, **** *p* < 0.0001; n.s.: not significant. (**C**) Separate panels reveal potential interactions between mouse genotype (WT/diabetic) and pressure condition (AP/NP) on temporal expression patterns. Binomial tests (probability = 0.25) evaluated peak time consistency across healing stages, with peaks at 16 h (DB AP), 2 h (DB NP and WT AP), and 0.5 h (WT NP). n = number of cytokine genes included in each stage.

The results, presented as box plots in Figure 2A and as line graphs in Figure 2B, highlighted the differences in cytokine expression between the AP and NP groups over time. Interestingly, these findings contrasted sharply with previous studies on normal mice under NP therapy (Figure 2C and referenced data). In a high-glucose environment, NP therapy did not significantly induce cytokine expression related to coagulation. Clinically, this suggests that NP therapy for diabetic patients should be carefully managed with attention to coagulation-related care.

Moreover, when comparing previously published data from normal mice with the diabetic mice in our current study, inflammation-related cytokines were consistently higher in the diabetic group (Figure 2C and referenced data). In the high-glucose, inflammation-prone environment, NP therapy significantly reduced inflammation-related cytokines, albeit with a noticeable delay in timing (Figure 2B,C, and referenced data). This delay corresponded with the prolonged wound healing observed in diabetic mice.

### 2.3. Differential Cytokine Expression Patterns Under Negative Pressure Wound Therapy in Diabetic Mice

Next, we visualized the normalized fold changes in cytokine gene expression using a heat map, where each row represents a cytokine gene categorized by its respective wound healing stage. Z-scores were calculated separately for previously published data from WT mice and the diabetic mice in our current study, accounting for potential genotype differences and isolating variations due to pressure conditions and time. Cytokines within each stage were ordered based on their z-scores at 0.5 h in the diabetic NP group, highlighting genes with the most significant expression changes under this specific condition. The heatmap uses a red-to-blue gradient, with red indicating higher z-scores.

**Figure 3:**
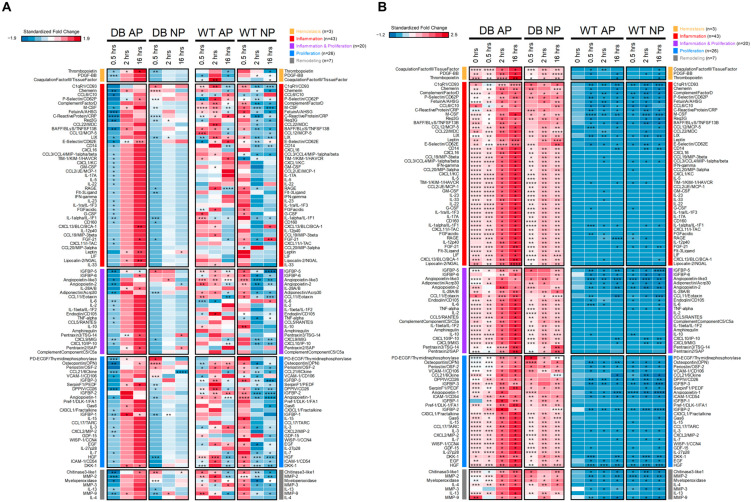
Coordinated regulation of cytokines by pressure and time. These heatmaps present standardized fold changes (z-scores) in cytokine gene expression. Each row represents a single cytokine gene, grouped by its wound healing stage. The baseline for fold change calculations was established using 0-h data from the same genotype (**A**) or WT AP (**B**). *p*-values for fold change significance were calculated by Student’s *t*-tests: * *p* < 0.05, ** *p* < 0.01, *** *p* < 0.001, **** *p* < 0.0001; otherwise: not significant. To account for potential genotype differences and isolate effects due to pressure and time, z-scores were calculated separately for WT and diabetic mice in (**A**). In (**B**), z-scores were calculated across both genotypes and 0-h data were included. Cytokines within each stage are sorted by their z-scores at 0.5 h in diabetic NP, highlighting the most differentially expressed genes under that specific condition. A red-blue color gradient represents z-scores, where red indicates higher values. n = number of cytokine genes included in each stage.

When the diabetic mice treated with AP were used as the baseline for comparison, and the results from previously published WT mice were included, an interesting pattern emerged (Figure 3A). In the comparison between diabetic AP and diabetic NP, NPWT consistently suppressed the expression of almost all cytokines, a stark contrast to the results previously observed in WT mice.

### 2.4. NPWT Effectively Modulates Inflammatory Cytokines

In diabetic mice, inflammatory cytokines are highly expressed two hours after wound formation, which is a key factor influencing poor wound healing in diabetes. Using large-scale cytokine arrays for simultaneous analysis and comparing with previously published data from WT mice, we observed that diabetic mice already show elevated levels of inflammation even without a wound, with all cytokine levels being higher than those in WT mice (Figure 3B). Interestingly, applying NPWT to the wounds in diabetic mice led to a significant overall reduction in these inflammatory cytokines, especially noticeable at the 2-h mark (Figure 3B).

### 2.5. Heightened Inflammatory State Delays NPWT-Mediated Wound Healing Modulation

**Figure 4:**
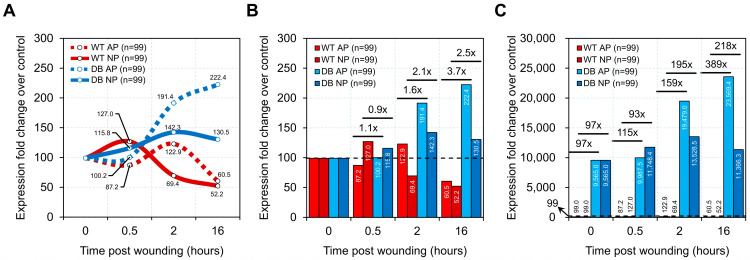
Mouse genotypes drive differential cytokine expression and pressure response. Diabetic mice exhibit higher total cytokine expression across all profiled genes, suggesting delayed wound healing and weaker response to negative pressure. Line charts (**A**) track changes over time, while bar charts (**B**,**C**) highlight differences at each time point for WT and diabetic mice. The baseline for fold change calculations was established using 0-h data from the same genotype (**B**) or WT AP (**C**). n = number of profiled cytokine genes. Dashed lines represent the baseline expression of WT AP (wild-type mice under atmospheric pressure) for visual reference.

In the comparison between diabetic and WT mice, using their respective 0-h time points as baselines, our previous research demonstrated that NPWT accelerates wound healing by modulating cytokine expression at each stage of the healing process [18]. However, in the highly inflammatory state of diabetic mice, this effect was significantly delayed by more than 12 h (Figure 4A). Interestingly, the difference between WT and diabetic mice approached a 100-fold disparity. In diabetic mice, inflammatory cytokines surged uncontrollably post-injury, reaching nearly a 200-fold increase at the 2-h mark and approximately a 400-fold increase by 16 h. In contrast, inflammatory cytokines in WT mice had returned to baseline levels by this time point (Figure 4B).

Although NPWT did not regulate wound healing in diabetic mice as effectively as in WT mice, it still exhibited a distinct modulation of inflammatory cytokines compared to AP treatment. By the 16-h time point, NPWT had begun to stabilize and reduce inflammatory cytokine levels in diabetic mice (Figure 4A). Overall, the maximum increase in inflammatory cytokine expression in diabetic mice reached up to 218-fold compared to WT mice (Figure 4B).

### 2.6. One-Sided Binomial Test Reveals NPWT Accelerates Wound Healing by Inducing Early Expression of Key Signaling Cytokines

**Figure 5:**
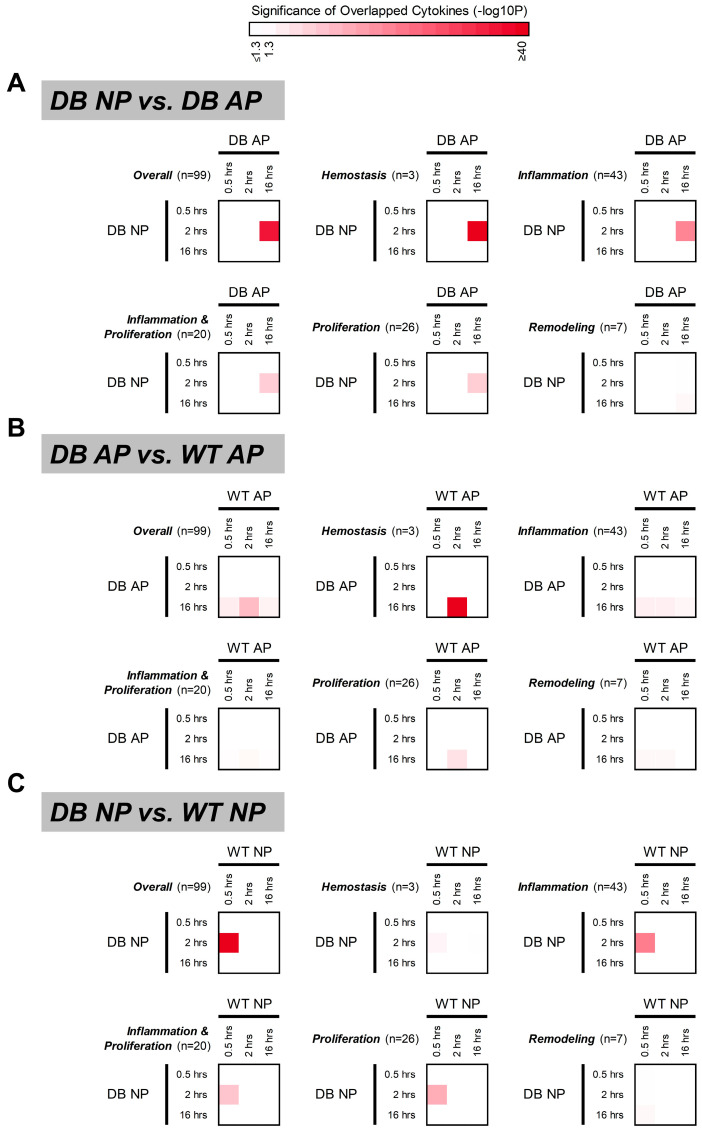
Concordance in peak cytokine expression. This analysis investigates whether similar sets of cytokines (overall/stage-specific) show peak expression at varying time points (0.5, 2, and 16 h) across different groups (genotype/pressure) following wounding. (**A**) Overlap in cytokines peaking at 2 h (NP) and 16 h (AP) suggests NP promotes early healing through similar cytokine regulation in diabetic mice. (**B**,**C**) Overlap in cytokines between genotypes suggests similar sets are involved in responses to AP (WT: 2 h vs. diabetic: 16 h) and NP (WT: 0.5 h vs. diabetic: 2 h), with WT mice exhibiting faster peak expression. Color gradient (log10 *p*-value): Darker red indicates stronger concordance (more significant overlap) in peak cytokines (binomial test; see Methods for details); white: not-significant (*p* ≥ 0.05). n = number of cytokine genes.

We employed a one-sided binomial test to determine whether specific cytokine probes exhibited peak expression at particular time points in diabetic mice treated with NPWT or AP. The analysis was conducted on the overall cytokine expression (Overall) and on each wound healing stage separately (hemostasis, inflammation, inflammation and proliferation, proliferation, and remodeling). This test assumed an equal probability (1/3) of peak expression occurring at any of the three profiled time points (0.5, 2, and 16 h). Interestingly, we found that the cytokine peaks in diabetic mice treated with NPWT at the 2-h mark matched those seen in AP-treated mice at 16 h (Figure 5A). When examining individual stages of wound healing, the hemostasis and inflammation stages showed a similar pattern to the overall cytokine expression trend (Figure 5A). However, due to the prolonged wound healing time in diabetic mice, peaks were less pronounced for the inflammation and proliferation and proliferation stages, and no discernible peaks were observed for the remodeling stage (Figure 5A).

**Figure 6:**
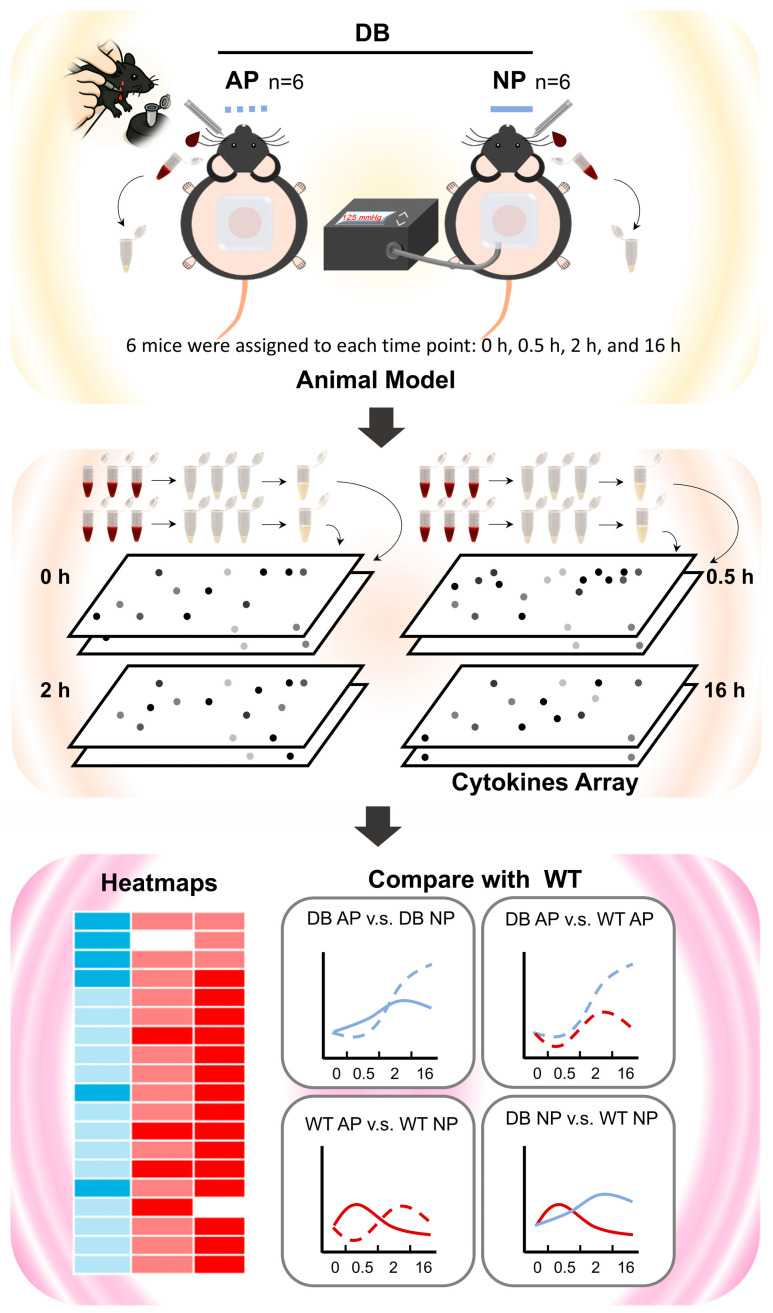
Overview of the experimental design and cytokine analysis. Diabetic (DB) mice were treated with atmospheric pressure (AP) or negative pressure wound therapy (NP), with serum collected at 0, 0.5, 2, and 16 h post-wounding for cytokine array analysis. Previously published wild-type (WT) data were used as a reference. Heatmaps and time-course plots visualize cytokine expression dynamics. A one-sided binomial test was applied to assess statistically significant shifts in cytokine peak timing between groups.

Next, we compared the effects of AP and NPWT on cytokine expression patterns in diabetic and WT mice. In diabetic mice, the heightened inflammatory state resulted in a significant delay in cytokine expression required for signal transduction, shifting the peak from 2 h to 16 h under AP treatment (Figure 5B). With NPWT intervention, this expression delay was attenuated, with the peak shift occurring from 0.5 h to 2 h (Figure 5C), indicating that NPWT partially restores early cytokine signaling and potentially accelerates wound healing in diabetic mice. In addition, the conceptual framework of the experimental design employed in this study is illustrated in Figure 6, whereas Supplementary Figure S1 provides photographic documentation of the dorsal wound sites in diabetic mice undergoing NPWT.

## 3. Discussion

Systematic reviews consistently demonstrate that NPWT accelerates wound healing and reduces complications in diabetic patients, particularly in preventing amputations related to DFUs [19,20,21]. Despite its clinical use, the precise molecular mechanisms of NPWT remain inadequately understood, especially during early wound healing. Diabetic wound healing is complex, involving various immune cells, cytokines, and growth factors [22,23,24].

In our study, we applied a novel time-stratified approach to profile cytokine expression at 0.5, 2, and 16 h post-injury in a diabetic mouse model. Our results confirmed elevated pro-inflammatory cytokine levels in diabetic wounds, contributing to prolonged inflammation and impaired healing. NPWT significantly modulated cytokine dynamics, reducing pro-inflammatory cytokines and promoting earlier activation of key signaling pathways involved in wound repair. Specifically, NPWT induced a marked temporal shift in cytokine expression, with peak levels of key cytokines such as IL-6 and TNF-α occurring at 2 h, compared to the control group (AP), which reached similar levels only at 16 h. This temporal adjustment suggests that NPWT not only accelerates the transition from the inflammatory to the proliferative phase but also mitigates the detrimental effects of prolonged inflammation commonly observed in diabetic wounds.

Furthermore, our findings align well with previously reported data by Arroba et al. [25], demonstrating similar trends of elevated cytokine expression, including TNF-α, IL-1β, IL-6, IL-13, IL-10, and IL-4, in BKS.Cg-Dock7m+/+Lepr^db/J mice. Notably, Arroba et al. compared db/db mice with heterozygous db/+ mice, whereas our study compared db/db mice with true wild-type (+/+) controls within the same strain background. Importantly, regardless of whether the comparison involved heterozygous db/+ controls or true wild-type (+/+) controls, the chronic inflammatory phenotype of db/db mice remained consistently evident, further reinforcing the reliability and biological relevance of our current observations.

In diabetic wounds, excessive expression of pro-inflammatory cytokines leads to prolonged inflammation, impairing the functions of macrophages and neutrophils, reducing the migration and proliferation of keratinocytes and fibroblasts, and diminishing the production of essential growth factors such as VEGF, IGF-1, TGF-β, and PDGF [26,27,28]. Growth factors play a pivotal role in regulating wound healing; however, hyperglycemia suppresses their synthesis and receptor expression, further complicating healing outcomes [29]. Reduced levels of VEGF and IGF-1 hinder neovascularization and granulation tissue formation [30,31,32]. Similarly, PDGF, a potent fibroblast mitogen, promotes connective tissue maturation and angiogenesis, but its expression is significantly reduced in diabetic wounds [29,33]. Basic fibroblast growth factor (bFGF) is known to accelerate granulation tissue formation and wound healing by stimulating mesodermal cell proliferation and extracellular matrix (ECM) synthesis. Clinically, bFGF enhances wound healing by promoting cell proliferation, migration, and differentiation while reducing scar formation [34,35,36]. Moreover, matrix metalloproteinases (MMPs) play crucial roles in wound debridement, angiogenesis, and ECM remodeling. In diabetic patients, MMP expression is upregulated, while tissue inhibitors of metalloproteinases (TIMPs) are downregulated, leading to excessive ECM degradation and impaired healing [37,38]. This imbalance prolongs the inflammatory response and disrupts collagen structure, ultimately delaying wound closure [39,40]. Mechanistically, RNA-binding proteins (RBPs), FUS and ILF2 may modulate keratinocyte function, reduce inflammation, and alleviate oxidative stress to promote DFU healing. Importantly, our findings suggest NPWT restores the balance between pro- and anti-inflammatory cytokines and enhances early growth factor activation, fostering an environment conducive to tissue regeneration.

Using a one-sided binomial test, we found that NPWT promotes early expression of key signaling cytokines, achieving peak levels at 2 h in diabetic mice, while the control group under AP only reached similar peaks at 16 h. This temporal shift indicates that NPWT reverses the typical cytokine response delay observed in diabetic wounds, potentially shortening overall healing time. Furthermore, NPWT’s regulatory effect on cytokine expression exhibited distinct patterns across different wound healing stages (hemostasis, inflammation, proliferation, and remodeling), underscoring its multifaceted role in managing diabetic wounds.

Clinically, NPWT has been widely applied to treat complex wounds such as diabetic foot ulcers and postoperative incisions. Its benefits include reduced edema, increased local perfusion, and accelerated granulation tissue formation. NPWT is also believed to enhance capillary permeability, facilitating the delivery of cytokines such as IL-10 and IL-8 to the wound bed, thereby promoting neovascularization [41]. However, due to the difficulty of capturing early molecular events in clinical settings, the precise mechanisms underlying NPWT’s early effects remain poorly understood. To address this gap, we employed a full-thickness wound model in diabetic mice and examined cytokine profiles at early time points, aiming to provide mechanistic insights that may guide the timing of early therapeutic interventions.

Nevertheless, certain limitations of the present study should be acknowledged. While our study centers on early-stage cytokine profiling, we recognize that cytokine changes represent only the initial stage of the wound healing process and are insufficient to reflect the full spectrum of functional recovery. In diabetic mice, complete wound healing generally requires more than seven days, and functional assessments such as histological analysis, wound closure rate, cellular proliferation, and fibrosis typically require extended observation periods. Previous studies have shown that NPWT enhances the expression of YAP and RhoA, suppresses the activity of En1- and CD26-positive fibroblasts, reduces the expression of α-SMA and HSP47, promotes cellular proliferation, and inhibits apoptosis—ultimately decreasing collagen deposition and improving scar quality [42]. Future investigations should incorporate long-term functional and histological analyses to develop a more comprehensive evaluation framework and elucidate the role of NPWT across all stages of wound healing.

## 4. Materials and Methods

### 4.1. Animals

Diabetic and obese male mice (genetically obese leptin receptor-deficient mice [db/db]; 7 weeks) were purchased from Jackson Laboratories (Bar Harbor, ME; Strain: BKS.Cg-Dock7^m^+/+ Lepr^db^/JNarl) and acclimatized in the Keelung Chang-Gung Memorial Hospital animal facility for 1 week before surgery. All procedures were performed in accordance with a protocol approved by the Institutional Animal Care and Use Committee (IACUC) of Chang Gung Memorial Hospital (IACUC-2020122226).

### 4.2. Surgical Procedure and Postsurgical Monitoring

Surgical procedures were performed on 8-week-old mice, which were randomly assigned to one of two treatment groups: the atmospheric pressure group (AP; traditional wet dressing) and the negative pressure wound therapy group (NPWT; −125 mmHg). Mice were evaluated at four-time points: 0, 0.5, 2-, and 16-h post-injury, with 3 mice used per group at each time point.

To ensure consistent application of negative pressure, mice were anesthetized with 4–5% isoflurane and positioned using a stabilizing apparatus (3% isoflurane). Analgesics and tranquilizers (diazepam: 5 mg/kg, administered intraperitoneally) were administered (ketorolac: 0.7–10 mg/kg, administered orally for 24 h) as needed to minimize discomfort. Dorsal hair was first clipped with electric clippers, followed by chemical depilation. A full-thickness excisional wound (6 mm in diameter) was then created on the dorsal skin using a biopsy punch (Kai Industries, Co., Ltd., Seki-shi, Japan).

To preserve wound geometry and prevent contraction, a 0.2 cm-thick artificial skin ring was placed around the wound perimeter. The wound was subsequently covered with a sterile dressing. For NPWT-treated mice, negative pressure (−125 mmHg) was continuously applied using the Apex NP therapy system for designated time intervals. Mice were euthanized at the assigned time points, and blood samples were collected for serum cytokine analysis.

This standardized mouse model effectively simulates full-thickness skin injury and allows controlled application of NPWT, enabling us to investigate the temporal effects of NP therapy on wound healing while minimizing procedural variability.

### 4.3. Cytokine Array Assay

Blood samples, approximately 500 µL each, were collected from the cheeks of mice following AP or NP treatment immediately prior to euthanasia. Samples were placed in 1.5 mL Eppendorf tubes and allowed to settle for 30 min to 2 h. The blood was then centrifuged at 1500× *g* for 10 min at 4 °C, after which the supernatant was collected and subjected to a second centrifugation at 13,000× *g* for 3 min at 4 °C to isolate the serum. Each experimental group (AP or NP) consisted of three mice, and 35 µL of serum from each mouse was pooled (a total of 105 µL), from which 100 µL was used for cytokine array analysis. Two cytokine array membranes (blots) were performed per group. Serum cytokine levels were measured using the Mouse XL Cytokine Array (R&D Systems, Inc. a Bio-Techne Brand McKinley Place NE Minneapolis, MN 55413, USA) following the manufacturer’s protocol. The arrays were processed using a UVP imaging system with images captured every 3 min over a 10-min exposure period. Quantification of the results was performed using ImageJ software, version 1.53e (JAVA 1.8.0, USA).

### 4.4. Cytokine Array Data Processing and Analysis

To account for variations arising from biological and technical sources across replicates within a single pressure condition (atmospheric or negative) and time point (0, 0.5, 2, or 16 h), a well-established array normalization method was employed [43]. Each array contained two replicates, with each replicate incorporating three reference spots (positive controls) for normalization purposes. These reference spots were used to calculate a total positive control intensity by summing the intensity values across all three spots. This value served as a reference point for normalizing the intensities of other cytokine probes on the array. The replicate (i) of the highest total positive control intensity served as the baseline, and each probe’s intensity on another replicate (j) was normalized to it by multiplying a factor of $T_{i}/T_{j}$, where $T_{i}$ and $T_{j}$ are the total positive control intensity of replicate i and j, respectively. The underlying assumption of this method is that the reference spots exhibit consistent intensity across replicates. WT cytokine array data (GEO: GSE252915) was downloaded and processed identically to the diabetic arrays to facilitate direct comparison between both datasets.

Fold changes for cytokine probes were determined by calculating the ratio of the normalized intensity for a specific probe in an experimental condition (AP or NP at 0.5, 2, or 16 h) to the control group (AP at 0 h). Each cytokine probe was further categorized into one of five functional groups reflecting wound healing stages, progressing from early to late: hemostasis (3 genes), inflammation (43 genes), inflammation and proliferation (20 genes), proliferation (26 genes), and remodeling (7 genes) [18]. To calculate the total fold change for each functional group, the fold changes of all member cytokines within that group were summed. This same approach was used to determine the total fold change for all cytokine probes on the array.

A one-sided binomial test was applied to investigate whether cytokine probes exhibited concordant peak expression within a specific time point combination (Figure 5). The test assumed an equal probability (1/3) of peak expression occurring at any of the three profiled time points (0.5, 2, and 16 h). We assessed the significance of concordant peak expression for both all cytokine probes collectively and for probes categorized by their specific wound healing stage. A *p*-value lower than 0.05 was considered statistically significant. The raw datasets are Supplemental Excel Files. 

## Data Availability

The original contributions presented in this study are included in the article. Further inquiries can be directed to the corresponding author.

## Supplementary Materials

The following supporting information can be downloaded at: https://www.mdpi.com/article/10.3390/ijms26104634/s1.

## Abbreviation

Atmospheric Pressure (AP); Basic Fibroblast Growth Factor (bFGF); Diabetic (DB); Diabetic Foot Ulcers (DFUs); Extracellular Matrix (ECM); Inducible Nitric Oxide Synthase (iNOS); Institutional Animal Care and Use Committee (IACUC); Matrix Metalloproteinases (MMPS); Negative Pressure (NP); Negative Pressure Wound Therapy (NPWT); RNA-Binding Proteins (RBPs); Tissue Inhibitors of Metalloproteinases (TIMPs); Wild-Type (WT); Chang-Gung Memorial Hospital (CGMH); Baylor College of Medicine (BCM); Research Associate (RA).
