# Supplementary material for: Temporal Regulation of Early-Stage Cytokine Expression in Diabetic Wound Healing Under Negative Pressure Wound Therapy
Source: Int J Mol Sci. 2025 May 13;26(10):4634. doi: 10.3390/ijms26104634 (PMC12110959; doi:10.3390/ijms26104634)
Supplement: Supplementary file 1 [file ijms-26-04634-s001.zip › Figure S1.pdf]

Figure S1

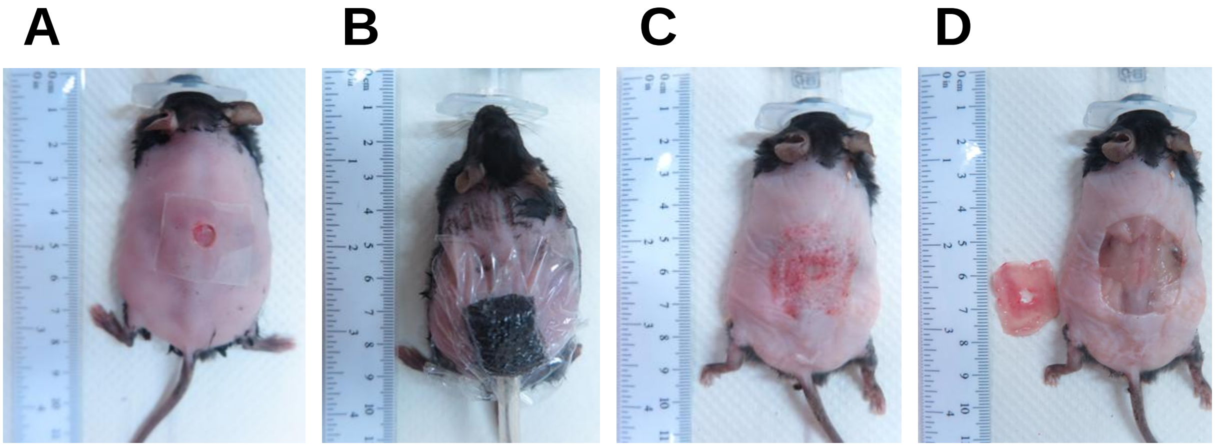

Figure S1. Photographic documentation of dorsal wound sites in diabetic mice under NPWT.
Each image includes a centimeter-scale ruler (left side) for reference.
(A) Photograph of the dorsal full-thickness wound site before treatment.
(B) Wound site under active negative pressure wound therapy (NPWT).
(C) Appearance of the wound immediately after NPWT removal.
(D) Excised skin surrounding the wound area, included for anatomical reference.
